# Translational Regulation of the DOUBLETIME/CKIδ/ε Kinase by LARK Contributes to Circadian Period Modulation

**DOI:** 10.1371/journal.pgen.1004536

**Published:** 2014-09-11

**Authors:** Yanmei Huang, Gerard P. McNeil, F. Rob Jackson

**Affiliations:** 1 Department of Neuroscience, Sackler School of Biomedical Sciences, Tufts University School of Medicine, Boston, Massachusetts, United States of America; 2 Department of Biology, York College, Jamaica, New York, New York, United States of America; Washington University Medical School, United States of America

## Abstract

The Drosophila homolog of Casein Kinase I δ/ε, DOUBLETIME (DBT), is required for Wnt, Hedgehog, Fat and Hippo signaling as well as circadian clock function. Extensive studies have established a critical role of DBT in circadian period determination. However, how DBT expression is regulated remains largely unexplored. In this study, we show that translation of *dbt* transcripts are directly regulated by a rhythmic RNA-binding protein (RBP) called LARK (known as RBM4 in mammals). LARK promotes translation of specific alternative *dbt* transcripts in clock cells, in particular the *dbt*-*RC* transcript. Translation of *dbt*-*RC* exhibits circadian changes under free-running conditions, indicative of clock regulation. Translation of a newly identified transcript, *dbt*-*RE*, is induced by light in a LARK-dependent manner and oscillates under light/dark conditions. Altered LARK abundance affects circadian period length, and this phenotype can be modified by different *dbt* alleles. Increased LARK delays nuclear degradation of the PERIOD (PER) clock protein at the beginning of subjective day, consistent with the known role of DBT in PER dynamics. Taken together, these data support the idea that LARK influences circadian period and perhaps responses of the clock to light via the regulated translation of DBT. Our study is the first to investigate translational control of the DBT kinase, revealing its regulation by LARK and a novel role of this RBP in Drosophila circadian period modulation.

## Author Summary

The CKI family of serine/threonine kinase regulates diverse cellular processes, through binding to and phosphorylation of a variety of protein substrates. In mammals, mutations in two members of the family, CKIε and CKIδ were found to affect circadian period length, causing phenotypes such as altered circadian period in rodents and the Familial Advanced Sleep Phase Syndrome (FASPS) in human. The Drosophila CKI δ/ε homolog DOUBLETIME (DBT) is known to have important roles in development and circadian clock function. Despite extensive studies of DBT function, little is known about how its expression is regulated. In a previous genome-wide study, we identified *dbt* mRNAs as potential targets of the LARK RBP. Here we describe a detailed study of the regulation of DBT expression by LARK. We found that LARK binds to and regulates translation of *dbt* mRNA, promoting expression of a smaller isoform; we suggest this regulatory mechanism contributes to circadian period determination. In addition, we have identified a *dbt* mRNA that exhibits light-induced changes in translational status, in a LARK-dependent manner. Our study is the first to analyze the translational regulation of DBT, setting the stage for similar studies in other contexts and model systems.

## Introduction

The Drosophila *doubletime* (*dbt*, a.k.a. *discs overgrown*, *dco*) gene encodes a protein homologous to human casein kinase I isoforms (CKI) [1], [2], in particular CKIδ and CKIε [3] . It is known that the DOUBLETIME (DBT/CKIδ/ε, hereafter referred to as “DBT”) kinase regulates cell proliferation, differentiation and cell polarity by functioning in Wnt [4], [5], Hedgehog [6]–[9], Fat [10]–[13] and Hippo signaling [14], [15] pathways. Those studies demonstrated roles of DBT in growth, development, organ size determination, and tumor suppression. The kinase is also well known for its role in the core molecular mechanism of the circadian clock ([1], [2], reviewed in [16]–[18]).

The molecular oscillator regulating locomotor activity rhythms is comprised of a transcription-translation feedback loop wherein accumulation of clock proteins regulates clock gene transcription and protein production. Transcriptional mechanisms are common to the circadian clocks of organisms ranging from cyanobacteria and fungi to plants and animals [18]–[22], although recent studies have indicated that conserved non-transcriptional clocks mediate certain types of circadian rhythms [23]. Casein kinase I (CKI) is required for period determination in vertebrates as well as insects. For example, in hamster and mouse, a gain-of-function mutation of CKIε (*CKIε^tau^*), causes shortening of circadian period [24], [25] whereas inhibition of CKIδ kinase activity in zebrafish disrupts circadian rhythmicity in locomotor activity [26]. In humans, a mutation in the key clock protein PERIOD 2 perturbs its phosphorylation by CKIε and is associated with Familial Advanced Sleep Phase Syndrome (FASPS), as a result of an abnormally short circadian period [27]–[31]. Interestingly, mutations in CKIδ were also found to cause FASPS in humans [32].

In Drosophila, the role of DBT in circadian period determination has been studied extensively. DBT was first shown to regulate PER accumulation [2], introducing a cytoplasmic lag into the circadian molecular loop. It was later established that DBT promotes progressive phosphorylation of PER, which facilitates interaction between PER and Slimb, an F-box/WD40-repeat protein that helps target PER for degradation in the proteasome [33]–[35]. Many DBT phosphorylation sites in the PER protein have been mapped [36]–[38]. Phosphorylation of residues in the so called “short-period domain” by DBT, gated by phosphorylation of a key residue by another kinase called NEMO/NLK, affects progression of the molecular cycle [39]. Phosphorylation of an N-terminal serine residue (S47) by DBT was identified as a key step in controlling the speed of the clock [40]. DBT is also required for phosphorylation of CLOCK (CLK), another key component of the Drosophila molecular clock [41], [42], although it was later found that DBT does not phosphorylate CLK directly but rather plays a non-catalytic role in CLK phosphorylation [43].

Despite extensive studies of DBT function, the mechanisms regulating expression of this protein are largely unknown. In a previous genome-wide study we identified *dbt* mRNA as a potential target of the LARK RBP, which has been implicated in translational control and clock function [44]–[52]. This suggested the possibility that *dbt* might be translationally regulated by LARK. Here we describe a detailed study of DBT regulation by LARK. We demonstrate that LARK can bind to and enhance translation of different transcript isoforms of *dbt* in clock cells of the adult fly head. The effect is most prominent with *dbt* transcripts *RC* and *RE*. Translation of *dbt*-*RC* undergoes circadian changes in free-running conditions, whereas translation of *dbt*-*RE* is light inducible. Consistent with the known role of DBT in circadian period determination, altered LARK expression in the PDF neurons affects period length, and this effect can be modified by *dbt* mutations. The role of LARK in modulating circadian period through DBT is further supported by the observation that increased LARK expression delays nuclear degradation of the PERIOD clock protein. Our study is the first to examine translational regulation of the DBT kinase and it supports a role of LARK in the modulation of circadian period.

## Results

### LARK binds *dbt* transcripts with high affinity

In a previous genome-wide study, we showed that *dbt* mRNA, but not other clock mRNAs, was associated with LARK *in vivo* [44]. The *dbt* gene produces multiple alternatively spliced transcripts. Earlier versions of genome annotation provided by FlyBase (up to Release 5.30) show three splice variants – *dbt*-*RA*, *dbt*-*RB*, and *dbt*-*RC* – that share protein-coding and 3′UTR sequence but differ at the 5′UTR (Figure S1A). However, the most recent annotation (release R5.49) included a fourth transcript, *dbt*-*RD*, that appears to be identical to *dbt*-*RB* but with a longer 3′UTR (Figure S1B). This difference is presumably based on recent genome-wide RNA sequencing data that includes sequence reads mapping to regions that extend beyond the previously annotated 3′UTR. However, we do not believe there is sufficient evidence to distinguish transcript *D* from transcript *B*; i.e., there may be only one transcript with a long 3′UTR. Thus we did not treat *dbt-RD* as an independent transcript, but instead focused our studies on the *dbt RA*, *RB* and *RC* transcripts. In addition, we found EST evidence suggesting the existence of an unannotated transcript with a unique 5′UTR, likely resulting from an alternative transcription start site. Two ESTs (GenBank gi 49381530 and gi 103690325) align perfectly to the 5′ region of the gene in a manner distinct from all previously annotated transcripts. We named this previously unannotated transcript *dbt-RE*. Studies described below demonstrate the expression of this novel transcript.

**Figure 1:**
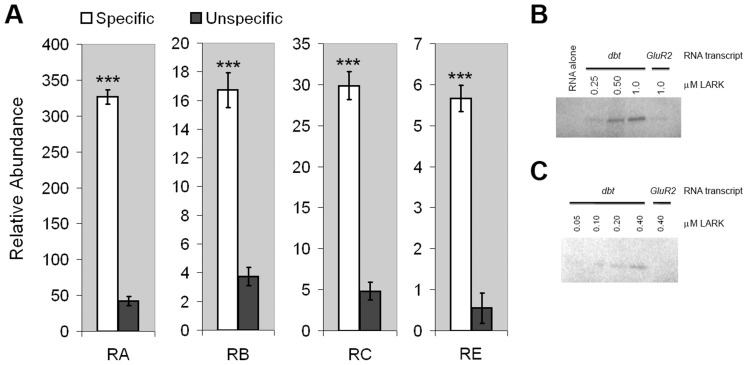
LARK associates with *dbt* transcripts. A, *dbt* transcripts specifically co-immunoprecipitate with LARK from head tissue lysates. Relative amounts of isolated RNAs using anti-LARK (specific) or anti-EGFP (unspecific) antibodies are shown for the four alternative transcripts of *dbt*. For each transcript, the mean value of at least 6 Q-RT-PCR experiments is shown. Error bars represent SEM. *** p<10^−6^ (Student's t-test). B and C, Purified recombinant LARK protein binds to *in vitro*-transcribed *dbt* RNA in UV cross-linking assays. RNA transcribed from *GluR2*, an unrelated gene, was used as a negative control. Two independent cross-linking assays, utilizing different LARK concentrations, are shown.

To determine if *dbt* transcripts are associated with LARK, *in vivo*, we quantified RNAs that co-immunoprecipitated specifically with LARK from head tissue lysates of adult flies. Quantitative Real-Time PCR (Q-RTPCR) using primers specific to each isoform demonstrated that *dbt* transcripts were enriched after anti-LARK immunoprecipitation (IP). Enrichment values, relative to transcript abundance after IP with an unrelated antibody (anti-EGFP) were 7.7, 4.5, 6.2 and 10.2 fold, respectively, for *dbt-RA, RB, RC, and RE* (Figure 1A). These results demonstrate an association between LARK and all *dbt* alternative transcripts *in vivo*.

These IP results do not distinguish between direct binding by LARK versus indirect association because of the presence of the RNA binding protein (RBP) and *dbt* mRNAs in the same complex. To test whether LARK can directly bind *dbt* mRNAs, we conducted UV cross-linking assays [53] using radio-labeled *dbt* transcripts produced by *in vitro* transcription (see Material and Methods) and a purified recombinant LARK protein containing both RNA Recognition Motifs (RRMs) [48]. This analysis showed that LARK binds to *dbt* mRNAs in a concentration-dependent manner and at concentrations as low as 100 nM (Figure 1, B and C). In contrast, LARK binding to an unrelated mRNA (*GlutR2*) was barely discernible at a concentration of 1 µM protein, indicative of specificity (Figure 1B). Thus, LARK can directly bind *dbt* mRNAs.

### LARK expression promotes translation of *dbt* mRNAs in clock cells and reveals a potentially new DBT isoform

**Figure 2:**
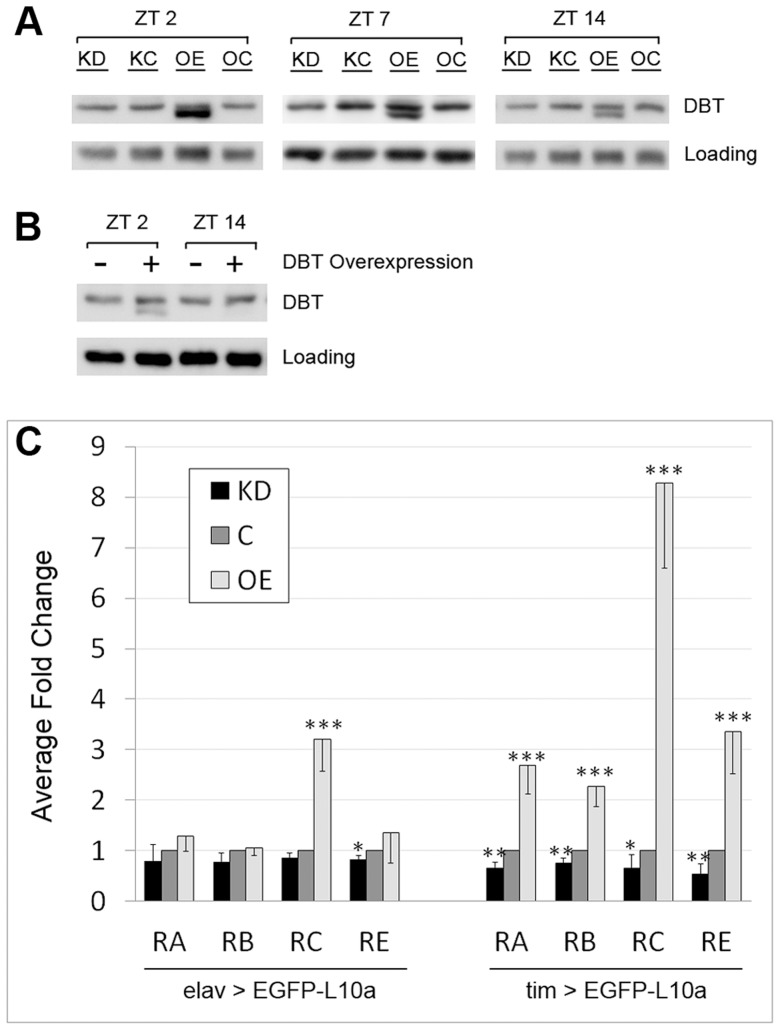
LARK regulates the translation of DBT transcripts. A, Western Blot showing the effect of pan-neuronal LARK knock-down (KD) and overexpression (OE) on DBT abundance assayed at three different zeitgeber times (ZTs). B, Overexpression of DBT alone renders detection of the shorter isoform at ZT2 but not ZT14. A non-specific band was used as loading control in A and B. C, Analyses of *dbt* mRNA translation using the TRAP method. All samples were collected at ZT2. Left, effect of altered LARK expression in all neurons. Right, effect of altered LARK expression in clock cells. Note that values for KD and OE are respectively normalized to KC and OC; thus the values of both controls were designated as “1” and plotted as one control denoted “C”. Fold changes were calculated from Ct values obtained from Q-RT-PCR that had been normalized to an internal Rp49 control. Average fold change from at least 6 Q-RT-PCR experiments are shown. Error bar represents SEM. *** p<0.001, ** p<0.01, * P<0.05 based on Student's t test comparing the Rp49-normalized Ct values of KD versus KC and OE versus OC.

To test the hypothesis that LARK regulates translation of the DBT protein, we examined the effect of altered LARK expression on DBT abundance. To our surprise, pan-neuronal overexpression of LARK (in *elav-gal4; uas-lark/+* flies) revealed a novel immunoreactive DBT band that was of lower molecular weight than the previously described protein (Figure 2A). To our knowledge, such a DBT immunoreactive protein has not previously been reported. In our experiments, however, the novel DBT band was consistently observed in all LARK overexpression (OE) samples but never in control (OC) samples. Furthermore, the band was detected at three different zeitgeber times (ZTs): ZT2, ZT7 and ZT14. We note that higher molecular weight bands are also detected by the DBT antibody (Figure S6) with LARK or DBT OE (seen with DBT OE on a longer exposure). As these bands are too big to represent single proteins encoded by *dbt* mRNAs and only seen with LARK or DBT OE, we think they must represent aggregates of DBT (see Discussion).

It is possible that the novel smaller DBT band represents an isoform that, in the absence of increased LARK expression, is normally present at a low undetectable level. To test this idea, we examined head tissue lysates of *elav-gal4*; *uas-dbt*/+ flies, which overexpress DBT in all neurons. We found that the novel protein was revealed by DBT overexpression (Figure 2B), indicating that it may represent a rare isoform of the protein. Interestingly, this novel isoform exhibits a diurnal oscillation: in LARK OE flies, it is more abundant at ZT2 than at ZT14 (Figure 2A). Similarly, in DBT overexpressing flies, it can be detected at ZT2 but not ZT14 (Figure 2B). In contrast to LARK OE, LARK knockdown (KD) does not produce a detectable effect on DBT protein level when assayed by Western analysis (Figure 2A). We attempted to show that the novel DBT band corresponded to a previously uncharacterized isoform of the kinase by examining null *dbt* mutants that survive to larval and early pupal stages (adult null mutants do not survive). However, LARK overexpression at these stages did not induce the novel band (Figure S7). Thus, it may represent an adult-specific form of DBT.

To directly assess the effect of altered LARK expression on translation of DBT, we used the Translating Ribosome Affinity Purification (TRAP) technique to isolate Ribosome bound RNAs from LARK OE, KD and the respective control flies (OC and KC). The TRAP technique was originally developed in mouse [54]. We and others have adapted the technique for use in Drosophila by constructing transgenic flies carrying a *uas-EGFP-L10a* construct that expresses EGFP-tagged ribosomes in target tissues when crossed to a GAL4 line; this permits isolation of translating mRNAs from target tissues [55], [56]. As LARK is known to have a pan-neuronal expression pattern in the adult head [4], we first generated flies with altered LARK expression in all neurons using *elav-gal4* in combination with *uas-lark^RNAi^* (for KD) or *uas-lark* (for OE). As indicated previously, knockdown or overexpression of wild-type LARK using these UAS constructs is associated with altered circadian behavioral rhythmicity [49], [51]. We included the *uas-EGFP-L10a* transgene in the OE or KD flies to allow isolation of translating mRNAs from all neurons. We found that LARK OE or KD did not significantly affect translation of *dbt-RA, RB or RE*. However, translation of *dbt-RC* was significantly increased in these experiments (Figure 2C, left) by LARK OE. Based on the knowledge that LARK and DBT both have circadian functions, we next examined the effect of altered LARK level on the translation of *dbt* transcripts in clock cells. In these experiments, we expressed *uas-lark* and *uas-EGFP-L10a* in clock cells using the *tim-uas-gal4* driver [57]. In contrast to pan-neuronal LARK OE, overexpression specifically in clock cells promoted translation of all *dbt* transcripts, with the effect on *dbt-RC* being the most dramatic (8 fold increased; Figure 2C, right). LARK KD caused a small but statistically significant decrease in the translation of all transcripts. To test whether the translational changes result from altered abundance of *dbt* transcripts or changes in translational status, *per se*, we examined *dbt* transcript levels in total RNA extracted from control and LARK OE flies. We found that overexpression of LARK in all clock cells of the fly head did not significantly affect the abundance of *RA*, *RB* or *RE* in total RNA samples. However, there was an approximate 2.6 fold increase in *RC* abundance (Figure S2). Such an increase in abundance cannot account for the observed 8.3 fold increase in translation of *RC* (Figure 2C, right). Thus, it is likely that LARK OE results in changes in *dbt-RC* translational status.

Taken together, the results of these experiments demonstrate that LARK promotes translation of DBT, in particular a previously unidentified DBT isoform. The observation that LARK expression in clock cells had more dramatic effects on *dbt* than pan-neuronal expression of the protein suggests that regulation of *dbt* translation by LARK may occur predominantly in clock neurons. An alternative but less likely explanation is that *tim-uas-gal4* drives higher expression of LARK than *elav-gal4*. However, we observed a similar level of expression for the two drivers when they were used with a *uas-GFP* reporter transgene.

### Circadian or diurnal changes in the translation of two low-abundance *dbt* transcripts

**Figure 3:**
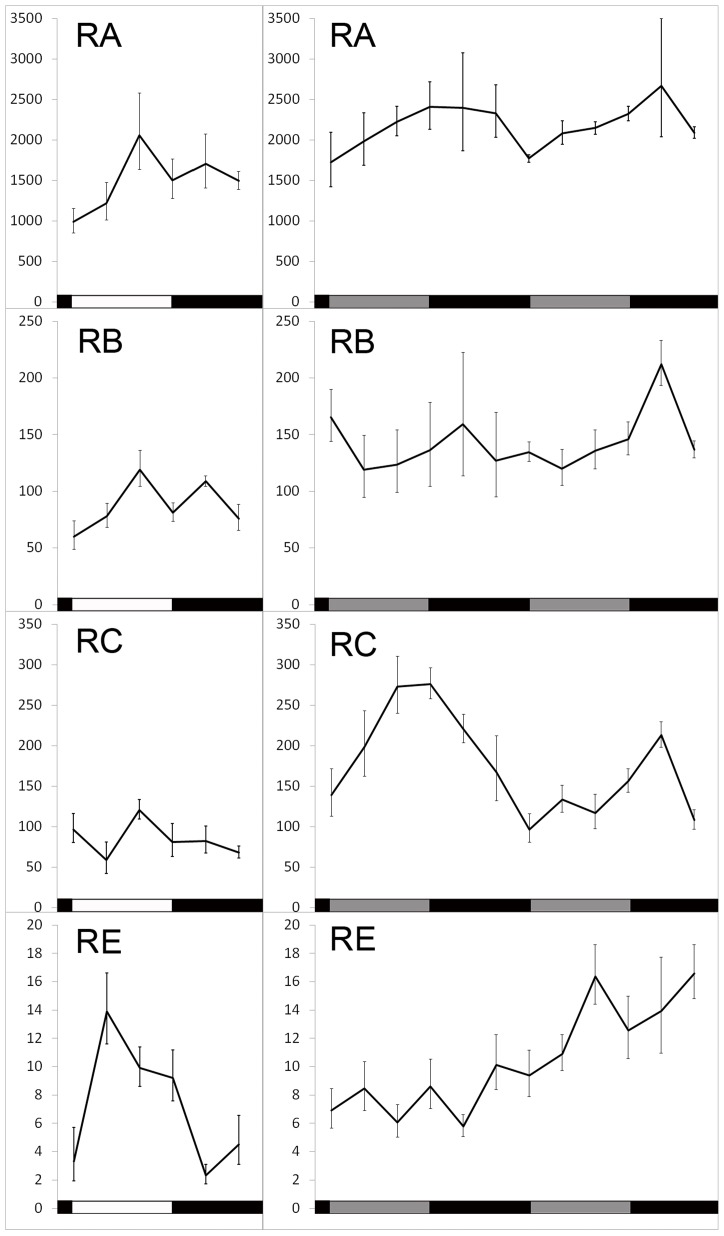
Translation of *dbt* transcripts in LD 12∶12 or constant dark (DD) conditions. Ribosome-bound RNAs were captured by TRAP at indicated Zeitgeber or circadian times and quantified by real-time Q-RT-PCR. Left panel, samples were collected over the course of one day. Right panel, samples were collected during the first and second day of DD. Mean and SEM (error bar) values for at least 6 Q-RT-PCR experiments are shown. One way ANOVA shows a time-dependent change of RC translation in both DD1 (p = 0.036) and DD2 (p = 0.00028), as well as RE translation in LD (p = 0.026).

In wild-type flies, LARK shows a circadian oscillation in abundance; the level of LARK is high during the day and low at night [47]. If LARK promotes translation of DBT, then the translational profile of DBT might also display a circadian rhythm. To test this hypothesis, we sampled the translational profiles of the four different *dbt* transcripts at 4-hour intervals under entrained conditions (LD 12∶12) and during the first 2 days of free-running conditions (DD). We emphasize that the endogenous LARK level was not manipulated in these experiments. We found that translation of *dbt-RA* displayed a low-amplitude rhythm in LD (peak to trough change is ∼2 fold), whereas *dbt-RB* and *dbt-RC* did not display rhythmic changes in translation. In contrast, *dbt-RE* displayed robust diurnal changes, with an 8-fold difference between trough-to-peak levels in LD (Figure 3, left panel; p = 0.036). The rhythms of *RA* and *RE* were greatly damped or eliminated when flies were released into free-running conditions (DD1 and 2). Interestingly, translation of *dbt-RC* appeared to begin cycling in DD, with a trough-to-peak change of about ∼2–3 fold (Figure 3, right panel; DD1, p = 0.036, DD2, p = 0.0003). *dbt-RB* translation did not exhibit significant rhythmic changes in LD or DD (Figure 3). Previous studies of total RNA extracted from whole adult head did not find significant circadian cycling of the *dbt* messages [1], [58], although Abruzzi *et al.* reported a low-amplitude cycling of *RC* in LD that did not reach their cutoff (1.4 fold change) for statistical significance. In agreement with those studies, we did not find significant cycling of the *dbt-RC* transcript in DD1 or *dbt-RE* in LD when abundance of these transcripts was examined in total RNA extracted from the same head lysate used in the TRAP assay (Figure S3). We conclude that *RE* and *RC* exhibit translational cycling in LD and DD, respectively.

### Light induced translation of *dbt-RE*

**Figure 4:**
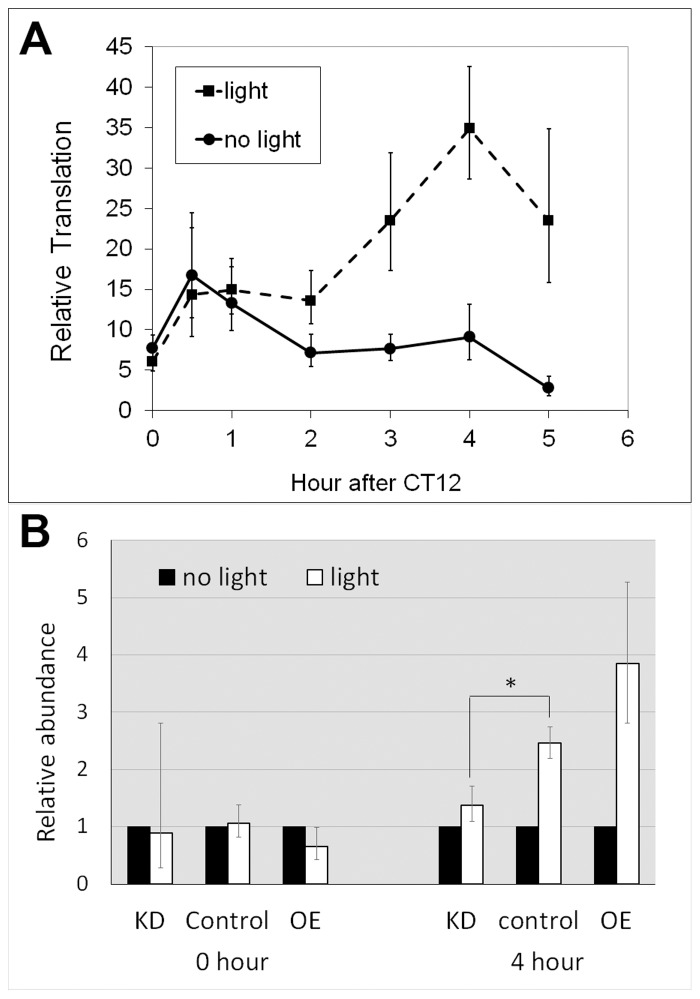
Altered LARK expression affects light-induced translation of *dbt-RE*. A. Light-induced translation of *dbt-RE* in wild-type flies. Relative translational levels were analyzed by quantifying ribosome-associated transcripts using TRAP and Q-RT-PCR. n≥5 for all data points. Error bars represent SEM. p = 2.91×10^−5^ analyzing the effect of light exposure by a two-way ANOVA of light condition and time. B. Altered LARK expression affects light-induced translation of *dbt-RE*. Light-induced translation of *dbt-RE* in flies with different LARK levels (KD, Control and OE) were analyzed by TRAP and Q-RT-PCR immediately after light exposure (0 hour) and 4 hours after light exposure. Amounts of ribosome-associated *dbt-RE* in flies exposed to light were normalized to those in flies kept in darkness (no light). n = 6 for all groups, representing 3 biological replicates, each with 2 technical replicates. Error bars show the possible range of fold change calculated based of the SEM of the QPCR data. * p<0.031 (Student's t test).

The observations that translation of *dbt-RE* displays a robust cycle under LD but not DD, and that peak translation occurs shortly after lights-on suggest that its translation might be induced by light. To test this hypothesis, we entrained *tim-uas-gal4; uas-EGFP-L10a* flies for 4 days under LD 12∶12 conditions and then released them into constant darkness (DD) on the fifth day. During the first day of DD, the flies were divided into two groups; at CT12 (i.e. the beginning of subjective night) one group received light stimulation while the other was maintained in darkness. We then performed TRAP analysis using head tissues from the two groups of flies and examined translation of *dbt-RE* at 0.5, 1, 2, 3, 4 and 5 hours after CT12. As shown in Figure 4, translation of *dbt-RE* steadily increased, peaking at 4 hours following light exposure. In contrast, translation of *dbt-RE* remained relatively unchanged in the control group not exposed to light (Figure 4A). Statistical significance of the result was verified by a two-way ANOVA, which revealed light exposure as a factor influencing changes in translational level (p = 2.91×10^−5^). Together with the observation that *dbt-RE* abundance does not cycle in total RNA, this experiment strongly suggests that translation of *dbt-RE* is induced within clock cells of the adult head by light exposure.

We next examined whether the light-induced translation of *dbt-RE* is affected by altering LARK expression. We asked this question by comparing differences in ribosome-bound *dbt-RE* levels between flies receiving light stimulation at CT12 (the beginning of subjective night) and those maintained in constant darkness. Ribosome-bound RE transcript was examined in LARK knockdown, LARK OE and control flies at CT12 and CT 16, with or without light stimulation. Relative to controls and LARK OE, LARK knockdown flies had significantly decreased light-induced *RE* translation (Figure 4B). These results support a role for LARK in the light-induced regulation of *dbt-RE*.

### Altered LARK expression affects circadian period

**Figure 5:**
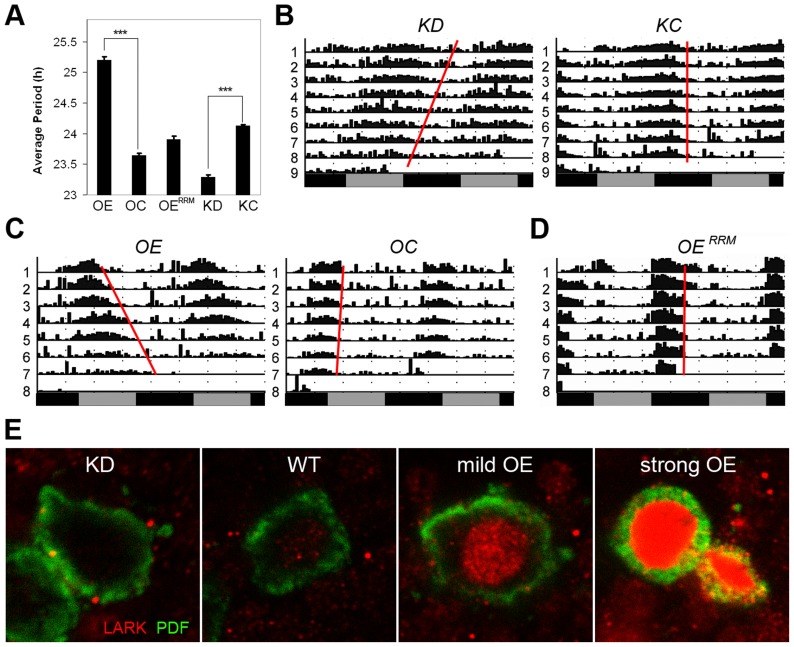
Altered LARK expression in PDF neurons affect circadian period. A, Average period length for various genotypes (n = 192, 144, 64, 130 and 166 for OE, OC, OE^RRM^, KD, and KC, respectively). Error bars represent SEM. *** p<10^−56^ between OE and OC; P<10^−57^ between KD and KC (Student's t-test). B–D, Representative activity plots for various genotypes. E, Immunohistochemistry showing LARK in the PDF neurons of various genotypes. KD, knockdown of LARK in PDF neurons, genotype: *w^1118^*; *pdf-gal4 uas-dicer2/+; lark^1^ uas-lark^RNAi^/+*. KC, control line for the knock-down, genotype: *w^1118^*; *pdf-gal4 uas-dicer2/+*. OE, moderate overexpression of LARK in PDF neurons, genotype: *w^1118^*; *pdf-gal4/+; Tub-gal80^ts^ uas-lark/+*. OC, control for the overexpression, genotype: *w^1118^*; *pdf-gal4/+; Tub-gal80^ts^/+*. OE^RRM^, Overexpression of a mutant form of LARK with defective RRM domains, genotype: *w^1118^*; *pdf-gal4/uas-lark^RRM^*.

The DBT kinase regulates PER phosphorylation and period of the circadian clock. Mutations that affect DBT level or its kinase function are known to alter period length of locomotor activity rhythms [1], [2], [59]. Given the observed effects of LARK expression on *dbt*, we tested whether alterations of LARK affect circadian period. We employed fly strains carrying a *uas-lark^RNAi^* transgene [51] for selective knockdown of LARK in specific subsets of neurons. This transgene was expressed throughout development, because we have not been successful in producing an adult-specific knockdown of LARK [50]. In order to achieve a more effective knockdown, the RNAi transgene was expressed in a background heterozygous for lark^1^, a null mutation of the gene [45]. As shown in Figure 5 (A and B) and Table S1, knockdown of LARK in the PDF neurons – important circadian pacemaker cells – caused an approximate 0.85 h shortening of circadian period. This effect is caused by specific knockdown by LARK, because the introduction of a *uas-lark* transgene into the LARK KD background reverted the period shortening (Figure S4, Table S1). Further, the effect is likely to be mediated by DBT because the period shortening was also corrected by introducing a *uas-dbt* transgene (Figure S4, Table S1). Predictably, conditional, adult-specific overexpression of LARK had the opposite effect, causing a 1.5 h lengthening of period (Figure 5, A, C, Table S1). It is of interest that LARK overexpression in this experiment caused period lengthening, because a previous study showed that conditional, high-level LARK overexpression, achieved using two copies each of *pdf-gal4* and *uas-lark* (Figure 5E, panel d), caused arrhythmic behavior [50]. We note that the present study utilized a “milder” level of LARK overexpression, achieved using only one copy each of *pdf-gal4* and *uas-lark*, revealing an effect on period. In addition, overexpression of LARK in this study was conditional and restricted to the adult stage, in contrast to a previous study which showed that mild overexpression of LARK throughout development caused increased arrythmicity rather than a lengthened period [49]. In the current study, the different levels of LARK OE and the effectiveness of LARK KD were validated by immunohistochemistry using anti-LARK antibody (Figure 5E). In contrast to wild-type LARK OE, a mutant LARK protein lacking function RRM domains [48], did not cause lengthening of period when overexpressed by *pdf-Gal4* (Figure 5, A, D, Table S1). We note that a previous study demonstrated that the UAS-wild-type and UAS-mutant *lark* transgenes are expressed at similar levels when driven by the same Gal4 driver [48]. These results indicate that the RNA-binding activity of LARK is required for the observed effects on behavior.

To confirm an effect on circadian period in LARK OE and KD flies, we looked at the cycling of PERIOD protein in the PDF neurons in conditions of constant darkness (DD). Abundance and localization of the PERIOD protein were examined every 4 hours for a 24-hour period by immunohistochemistry and confocal imaging. Because the period altering effects are small, especially in the case of LARK KD, we allowed the effect to accumulate for 4 days in DD. On day 4, the phase of the oscillator should have advanced by almost 4 hours in LARK KD flies, allowing the difference to become detectable when sampling every 4 hours. Indeed, we found that the phase of PER cycling is advanced in KD flies and delayed in OE flies (Figure S5), consistent with results of the behavioral analyses.

### *lark* genetically interacts with *dbt* to affect circadian period

**Figure 6:**
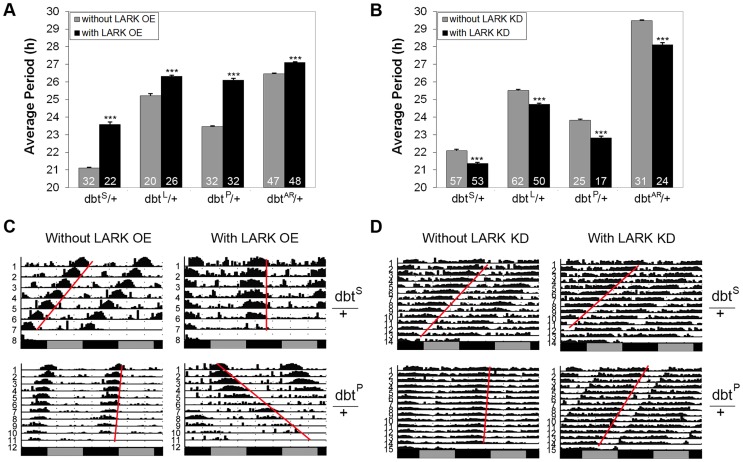
Interactions between *lark* and *dbt* modulate circadian period. A and B, Quantification of average period lengths showing effects of LARK OE or KD in flies heterozygous for various *dbt* mutations. Numbers shown at the base of the bar chart represent samples sizes for each genotype. Error bars represent SEM. p<0.0001 for all comparisons. C and D, Representative activity plots. For interactions involving LARK OE, genotypes are: without LARK OE, *w^1118^*; *pdf-gal4/+; Tub-gal80^ts^/dbt*. With LARK OE, *w^1118^*; *pdf-gal4/+; Tub-gal80^ts^ uas-lark/dbt*. For interactions involving LARK KD, genotypes are: without LARK KD, *w^1118^*; *pdf-gal4 uas-dicer2/+; +/dbt*, with LARK KD, *w^1118^*; *pdf-gal4 uas-dicer2/+; lark^1^ uas-lark^RNAi^/dbt* (*dbt* here refers to *dbt^S^*, *dbt^L^*, *dbt^P^*, or *dbt^AR^*).

To further test the possibility that LARK influences period length by modulating expression of DBT, we investigated genetic interactions between altered LARK expression and chromosomal *dbt* mutations including *dbt^L^*, *dbt^S^*, *dbt^P^* and *dbt^AR^*. We found that overexpression of LARK lengthened period in all the *dbt* mutant backgrounds tested. Interestingly, the period lengthening effect of LARK OE varied in different mutant backgrounds. The effect was more dramatic in mutants with short period than in mutants with long period. For example, overexpression of LARK caused a lengthening of ∼2.5 hour and ∼2.6 h, respectively, in the *dbt^S^/+* and *dbt^P^/+* backgrounds. In contrast, it caused only 1.1 and 0.63 h period lengthening in *dbt^L^* and *dbt^AR^* backgrounds (Figure 6, A and C). Such non-additive effects suggest a genetic interaction between *lark* and *dbt*. Similarly, knockdown of LARK caused period shortening in all *dbt* mutant backgrounds, with the effect being most prominent in a long-period background (*dbt^AR^/+;* Figure 6, B and D).

### DBT kinase activity is required to mediate the effect of LARK overexpression

**Figure 7:**
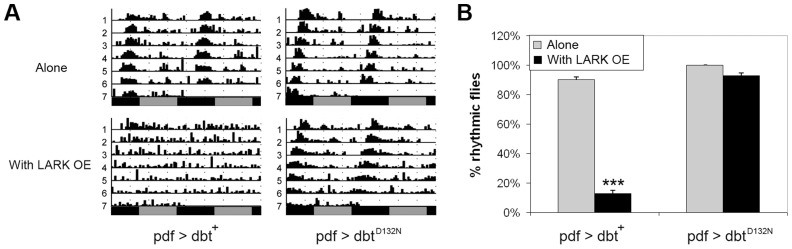
DBT kinase activity is required for the LARK OE phenotype. A: Representative actograms showing that overexpression of wild-type DBT protein (*pdf>dbt*) enhances the LARK OE phenotype (producing arrhythmicity) whereas overexpression of a mutant DBT protein lacking kinase activity (*pdf>dbt^D132N^*) suppresses the period-lengthening effect of LARK OE. B: Quantification of percentage rhythmicity in flies overexpressing DBT proteins with or without LARK OE. Genotypes are *pdf>dbt* alone: *pdf-gal4/+; uas-dbt/+* (n = 31). pdf>*dbt* with LARK OE: *pdf-gal4/+; uas-dbt/Tub-gal80^ts^ uas-lark* (n = 31). pdf>dbt^D132N^ alone: *pdf-gal4/+; uas-dbt^D132N^/+* (n = 42). pdf>dbt^D132N^ with LARK OE: *pdf-gal4/+; uas-dbt^D132N^/Tub-gal80^ts^ uas-lark* (n = 14). *** p<0.0001 by Chi-square test.

Our previous research found that high level LARK overexpression, using two copies each of *pdf-gal4* and *uas-lark*, resulted in complete arrythmicity [50]. Research by others has shown that overexpression of a wild-type form of DBT in clock cells has a minimal effect on period but causes a reduction in rhythmicity [60]. We asked whether the arrhythmic behavior caused by high-level LARK expression is mediated through DBT. To address this question, we generated *pdf-gal4/+; uas-lark/uas-dbt* flies that carry a single copy of each responder transgene. Such flies were arrhythmic compared to controls that only expressed the *uas-dbt* or *uas-lark* transgenes (Figures 5 and 7), indicative of an interaction between the genes. This interaction required DBT kinase activity, as overexpression of LARK and DBT^D132N^, a mutant form of DBT devoid of kinase activity [4] did not cause significant arrhythmicity (Figure 7). In contrast, overexpression of DBT^D132N^ suppressed the period-lengthening effect of mild LARK OE, possibly due to a dominant-negative effect caused by competition of the kinase-dead protein with wild-type protein. The average period for flies overexpressing LARK alone and flies overexpressing both LARK and DBT^D132N^ were 25.1±0.06 hours and 22.67±0.11, respectively (Table S1).

We note that a previous study by Muskus et al. (2007) showed that expression of a different kinase-dead mutation of DBT (DBT^K38R^) in clock cells caused a lengthened period or arrythmicity [60]. Thus, it is surprising that expression of DBT^D132N^ alone did not have obvious effects on period length or rhythmicity in our experiments (Table S1). However, Muskus et al drove expression of DBT^K38R^ in all clock cells throughout development using a *tim-gal4* driver. In this study we used the *pdf-gal4* driver to direct expression of DBT^D132N^ only in LNvs. More importantly, to avoid effects caused by potential developmental defects, we used the TARGET method [61] to confine expressing of DBT^D132N^ to adulthood. These factors may explain the differences between our observations and those of Muskus et al (2007).

### Increased LARK expression delays degradation of the PERIOD protein

**Figure 8:**
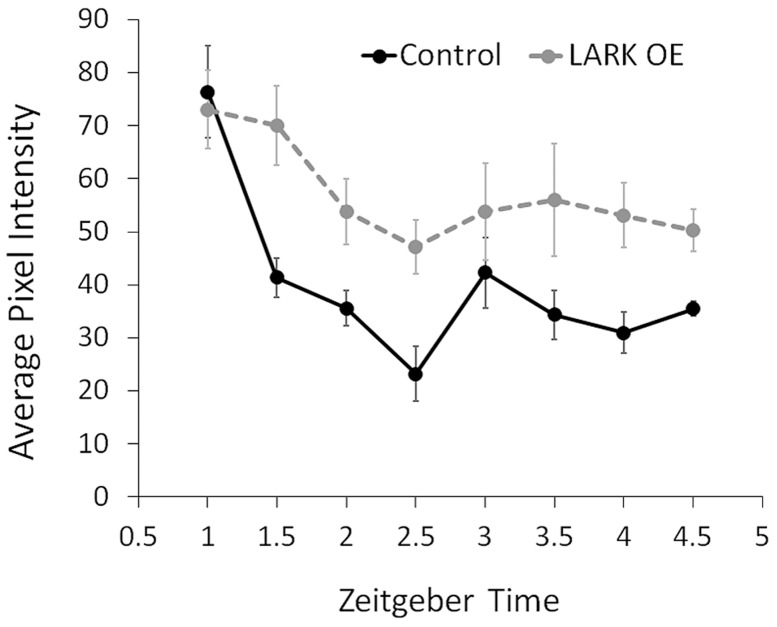
Increased LARK expression delays degradation of the PER protein. The average pixel intensity in confocal images of anti-PER immunoreactivity in large PDF neurons (l-LNvs) are quantified for control and LARK overexpression flies. Samples were taken every 0.5 hours starting at ZT1 till ZT4.5. For each data point, brain hemispheres from 4–6 different animals were analyzed. Error bar represent SEM. A two-way ANOVA of genotype and time find a significant difference between control and LARK OE (p = 0.006289).

DBT kinase is involved in multiple steps of the sequential phosphorylation of PERIOD, priming the clock protein for ubiquitin-mediated degradation (reviewed in [18]). PER degradation rate is a key determinant of circadian period length (reviewed in [18]). To test the possibility that LARK modulates period length by regulating DBT-dependent PER degradation, we monitored PER nuclear degradation in the PDF-positive large ventral lateral neurons (l-LNvs) by immunohistochemistry and confocal imaging. We found that LARK OE caused a reduced rate of PER degradation during the initial 2.5 hours after lights on in an LD cycle (Figure 8). This result suggests that LARK modulation of DBT results in altered PER degradation.

## Discussion

Despite many studies of DBT function in cellular signaling pathways and circadian period determination, little is known about the regulation of DBT itself. In this study we show that translation of *dbt* transcripts are regulated by a clock-controlled RBP called LARK. We provide direct evidence that LARK promotes the translation of *dbt* transcripts in clock cells. Western Blot analyses reveal a previously undescribed smaller isoform of DBT promoted by LARK overexpression (Figure 2). Although we could not examine this smaller protein in null mutants (see Results) - to show specificity of the DBT antibody - three observations suggest that it corresponds to a novel DBT isoform. First, LARK can bind to *dbt* transcripts and overexpression of the RBP promotes the appearance of the novel DBT immunoreactive band. Second, overexpression of *dbt*, similar to LARK, results in the appearance of the novel protein. Finally, the novel protein shows circadian changes in abundance that are in phase with those of LARK. Together, these findings indicate the existence of a novel DBT isoform, encoded by one or more *dbt* transcripts that are regulated by LARK.

As previously mentioned, LARK or DBT OE are associated with the appearance of higher molecular weight DBT immunoreactive bands in addition to the novel short isoform. (Figure S6). Individual proteins of these size classes cannot be encoded by known *dbt* mRNAs. Therefore, they likely represent aggregates of DBT. Their formation might be facilitated by interaction with the short isoform, which we postulate may act as a scaffold due to its lack of a kinase domain. Although we hypothesize that the short isoform is responsible for the period altering effect, our results do not rule out the possibility that these higher molecular weight complexes contribute to the observed phenotypes.

As demonstrated by Western analysis, the novel isoform has a slightly lower molecular weight compared to the known isoform of DBT, indicating a shorter amino acid sequence. Since the four alternative transcripts encode the same Open Reading Frame (ORF) and differ only in their 5′UTR, it is possible that binding of LARK promotes translation from an AUG, or an unconventional initiation sites such as CUG, GUG, UUG, or ACG, downstream of the conventional start codon. It is known that translation of another target of LARK, E74A, utilizes at least three alternative initiator codons: two minor forms of the protein are initiated at a CUG and an AUG, while the most abundant form initiates at a CUG [62]. Similar to DBT, our previous studies of E74A show that LARK overexpression dramatically increases E74A protein abundance, changing the level from barely detectable to very high [44]. Of note, the mammalian homolog of LARK, RNA Binding Motif Protein 4 (RBM4), is known to promote cap-independent, internal ribosome entry site (IRES)-mediated translation when phosphorylated by the p38 MAPK pathway [63]. It is possible that the smaller isoform of DBT results from IRES-mediated translation.

At present, we do not know which *dbt* transcript expresses the short DBT isoform although all four transcripts are capable of encoding it. We also note that our results do not rule out an alternative but unlikely possibility that LARK OE results in DBT proteolytic cleavage resulting in the smaller isoform. However, the observations that LARK binds *dbt* RNA and promotes ribosome association of dbt transcripts without causing a significant change in abundance of the larger DBT isoform indicates that LARK may promote translation of the small isoform.

As the conserved kinase domain of DBT starts close to the 5′ terminus at amino acid 15, any alternative initiation site downstream of the original AUG is likely to affect kinase activity. Thus, it is possible that the short DBT isoform has no kinase activity but rather plays a structural role. A non-catalytic role of DBT has been suggested by others in a recent study. Yu et. al. (2009) found that PER-DBT binding, but not DBT catalytic activity, is required for CLK hyperphosphorylation and transcriptional repression and proposed a model in which DBT plays a novel, noncatalytic role in recruiting additional kinases that phosphorylate CLK, thereby repressing transcription [36]. Our results indicate that both the LARK-induced short isoform and full length wild-type DBT are required to exert the period lengthening effect, as co-expressing a kinase-dead form of full length DBT abolishes the period-lengthening effect of LARK OE (Figure 7). These results suggest that the short-isoform and full-length kinase may interact to set the speed of the clock. A plausible hypothesis is that the short DBT isoform serves as a non-catalytic subunit which modulates full-length DBT kinase. Thus, the ratio of short to full-length DBT may be important for modification of PER.

In a previous genome-wide study we identified many mRNAs that are associated with LARK *in vivo* [44]. Among these LARK-associated mRNAs, only three others encode proteins that are known to be involved in circadian function: *flapwing* (*flw*), *no receptor potential A* (*norpA*), and *dunce* (*dnc*). We did not detect association of LARK with canonical clock mRNAs (*per, tim, clk, cyc, etc.*). Thus it seems likely that the effect of LARK on period is mediated by DBT.

How might LARK regulate DBT and circadian period? As already indicated, RBM4 (mammalian LARK) is activated and shuttles to the cytoplasm to regulate IRES-dependent translation in response to p38 phosphorylation [64]. Interestingly, evidence suggests that p38 may have roles in circadian clock function [65], [66], and it is known to mediate circadian output and/or clock responses to light in several systems [67], [68]. Thus, the known clock regulation of LARK [47] may, in part, depend on p38-mediated phosphorylation of the protein. In turn, changes in LARK amount or activity might regulate DBT translation, as suggested by our study.

Alterations in DBT expression are predicted to modulate circadian period, by affecting either the accumulation or degradation of PER. Our results show that PER degradation in clock neurons is prolonged, *in vivo*, by increased LARK expression (Figure 8).

PER degradation requires binding of SLIMB, an F-box protein that helps target proteins to the ubiquitin–proteasome degradation pathway [34], [35]; SLIMB binding to PER requires a series of sequential phosphorylation events on PER [40]. These include phosphorylation at S661 and residues within a so-called “per-short domain”, spanning amino acids S585 to Y601, to which mutations that shorten period have been mapped (*per^S^* and *per^T^*; [69]–[74]). Chiu et al. (2011) have shown that phosphorylation of the per-short domain by the NEMO and DBT kinases (including S589, a DBT target residue) slows down phosphorylation of PER S47, a critical event for binding of SLIMB and PER degradation [40]. Lack of per-short domain phosphorylation leads to faster degradation of PER and short-period behavioral rhythms [40]. These results are consistent with a previous study suggesting that the per-short domain regulates the activity of DBT against PER [75]. Thus, enhanced or prolonged phosphorylation of this domain may lengthen period. We postulate that increased LARK expression and production of a short, non-catalytic DBT isoform leads to delayed PER degradation and lengthened circadian period by altering the timing of DBT-mediated phosphorylation of the per short domain. The observation that *dbt^P^*, which is a hypomorphic allele of *dbt*, enhances the period lengthening effect of LARK OE (compare Figure 6 with Figure 5, also see Table S1) suggests that alteration of the short to full-length DBT ratio may be responsible for period lengthening. Interestingly, a mutant form of DBT (DBT^AR^) that was suggested to play a non-catalytic, auxiliary role – similar to our proposal for the DBT short isoform – also causes period lengthening in heterozygotes [75].

Our analysis of DBT regulation revealed a *dbt* transcript showing light-inducible translation that is affected by LARK levels (Figure 4). This novel transcript, *dbt*-*RE*, shows a translational oscillation that is in phase with LARK abundance in LD conditions and it can be induced by light in dark conditions. Together with the observation that LARK abundance is highest at the beginning of the day [47], these results suggest that this RNA-binding protein may be light inducible in addition to showing circadian variation. In LD conditions, the light-induced increase in LARK level may up-regulate translation of *dbt*-*RE*. Based on the observation that *dbt*-*RE* represents an extremely small fraction of all ribosome-associated *dbt* transcripts (∼0.56%) captured by TRAP, it is possible that such a light-induced event occurs only in a small number of adult head clock cells, perhaps only in cells that mediate the light response. Although a role for LARK and DBT in pacemaker light sensitivity has not been reported, our study suggests it may be of interest to explore this possibility.

## Materials and Methods

### Drosophila strains, rearing conditions and genetic crosses

The following stocks were obtained from the Bloomington Stock Center (stock number in parenthesis): *w^1118^* (5905), *elav-gal4* (458), *uas-dbt* (26269 and 26274) *dbt^P^* (12164) and *uas-dicer2* (24650). *uas-lark*, *uas-lark^RRM^* and *uas-lark^RNAi^* were described previously [49], [51]. *uas-EGFP-L10a* is a transgenic line generated in our lab that carries a UAS construct for expressing EGFP-tagged mouse ribosomal protein L10a [55]. *tim-uas-gal4* was obtained from Dr. Justin Blau, pdf-gal4 was obtained from Dr. Patrick Emery, *dbt^L^*, *dbt^S^*, *dbt^AR^* were provided by Dr. Paul Hardin, *uas-dbt^D132N^* was provided by Dr. Marek Mlodzik. Flies were raised in incubators set at 25°C and 60% humidity and a light-dark cycle consisting of 12 hours of light and 12 hours of dark (LD 12∶12) unless specified otherwise.

For Western Blot (Figure 2) experiments, genotyppes are: KD, *elav-gal4(/+); uas-dicer2/+; uas-lark^RNAi^/+*. KC, *elav-gal4(/+); uas-dicer2/+*. OE, *elav-gal4(/+); uas-lark/+*. OC, *elav-gal4(/+)*. DBT overexpression, *elav-gal4(/+); uas-dbt/+*. Control for DBT overexpression, *elav-gal4(/+); +/+*. Note that “*elav-gal4(/+)*” denotes the fact that male flies are hemizygous for *elav-gal4* and female flies are *elav-gal4/+*.

For TRAP experiments, genotypes for examining the effect of altered LARK expression in all neurons are: KD, *elav-gal4(/+); lark^1^ uas-lark^RNAi^/uas-EGFP-L10a*. C, *elav-gal4(/+); uas-EGFP-L10a/+*. OE, *elav-gal4(/+); uas-lark/uas-EGFP-L10a*. Genotypes for examining the effect of altered LARK expression in all clock cells are: KD, *w^1118^*; *tim-uas-gal4/+; lark^1^ uas-lark^RNAi^/uas-EGFP-L10a*. C, *w^1118^*; *tim-uas-gal4/+; uas-EGFP-L10a/+*. OE, *w^1118^*; *tim-uas-gal4/+; uas-lark/uas-EGFP-L10a* (Figure 2). The genotype for examining circadian (figure 3) or light-induced (Figure 4) translation of *dbt* transcripts is *w^1118^*; *tim-uas-gal4/+; uas-EGFP-L10a/+*.

For locomotor behavior assays, genotypes are: KD, *w^1118^*; *pdf-gal4 uas-dicer2/+; lark^1^ uas-lark^RNAi^/+*. KC, *w^1118^*; *pdf-gal4 uas-dicer2/+*. OE, *w^1118^*; *pdf-gal4/+; Tub-gal80^ts^ uas-lark/+*. OC, *w^1118^*; *pdf-gal4/+; Tub-gal80^ts^/+*. OE^RRM^, *w^1118^*; *pdf-gal4/uas-lark^RRM^*. *pdf>dbt* alone: *pdf-gal4/+; uas-dbt/+*. pdf>*dbt* with LARK OE: *pdf-gal4/+; uas-dbt/Tub-gal80^ts^ uas-lark*. pdf>dbt^D132N^ alone: *pdf-gal4/+; uas-dbt^D132N^/+*. pdf>dbt^D132N^ with LARK OE: *pdf-gal4/+; uas-dbt^D132N^/Tub-gal80^ts^ uas-lark*. To prevent developmental effects known to be caused by LARK OE, the crosses and progeny were reared at 23°C until the time of experiment, when they were transferred into 30°C to deactivate the protective effect of *Tub-gal80^ts^* and allow OE to be achieved. To examine genetic interaction between LARK OE or KD and various chromosomal mutations of *dbt*, virgin females from either the *w^1118^*; *pdf-gal4; uas-lark Tub-gal80^ts^* strain (for OE) or the *w^1118^*; *pdf-gal4 uas-dicer2; lark^1^ uas-lark^RNAi^*/*TM2 Ubx* strain (for KD) were crossed to males of the *dbt^L^*, *dbt^S^*, *dbt^P^*, or *dbt^AR^*, respectively, and male progeny of the crosses were used for the behavioral analyses.

### Co-IP assay

Polyclonal rabbit anti-LARK antibodies [47] were used for IP of LARK protein. A mono-clonal mouse anti-EGFP (clone 19C8 from MACF), was used as a control for unspecific bindings of RNAs to antibody-coupled Dynabeads. The antibodies were coupled to Dynabeads (Invigrogen) according to manufacturer's instruction. Flies of the *w^1118^* strain were entrained to LD 12∶12 for 3 days and then flash frozen in liquid nitrogen at ZT2. Heads were harvested and homogenized in a mild lysis buffer containing 100 mM KCl, 5 mM MgCl_2_, 10 mM HEPES PH 7.0, 0.5% Ipegal-CA630, 1 mM DTT, 1 mM PMSF, and 10 µg/ml protease inhibitor cocktail (Sigma). The homogenates were incubated on ice for 5 minutes and centrifuged at 14,000× g for 20 minutes at 4°C. Cleared lysates were incubated with antibody coupled Dynabeads at 4°C for 1 hour. Following incubation, the supernatants were removed and the beads were washed 6 times using a buffer containing 20 mM HEPES-KOH (pH 7.4), 5 mM MgCl_2_, 350 mM KCl, 1% IGEPAL-CA630, and 0.5 mM DTT. RNAs were extracted from the immunoprecipation using the Trizol LS reagent (Invitrogen) and reverse transcribed into cDNA using Superscript II reverse transcriptase (Invitrogen) with random hexamers. The various *dbt* transcripts in the anti-LARK immunoprecipitated and anti-EGFP immunoprecipitated samples were analyzed by Q-RT-PCR using primers specific to each transcript (see below).

### RNA binding assay

RNA transcripts used in the UV cross-linking assays were synthesized *in vitro* using ^32^P-UTP and the MEGAscript Kit (Ambion). The cDNA template for *dbt* was obtained from the Drosophila Genomics Resource Center (EST clone LD 27173) and for *GluR2* was obtained from Dr. Joel D. Richter. A LARK N-terminal GST fusion protein containing the N-terminal RNA-binding domains (two RRM domains and one RTZF) was synthesized and purified using the Pierce GST Purification Kit. RNA-protein binding reactions were carried out according to [53]. Briefly, 1×10^5^ cpm of *in vitro* synthesized RNA transcript and varying amounts of LARK-GST fusion protein were added to 2X GR buffer (20 mM HEPES, pH 7.6, 100 mM KCl, 2 mM MgCl_2_, 0.2 mM ZnCl_2_, 20% glycerol, 2 mMDTT), 10 ng t-RNA, 1.2U Rnase OUT (Life Technologies), and 1 mM DTT and incubated on ice for 10 min. followed by RT for 10 min. 50 mg of heparin was added to the mixture followed by UV exposure at 440 mJ for 3 min. RNase A (10 ng) was added and incubated for 30 min at 37°C. The products were resolved by SDS-PAGE and binding was detected using a Typhoon Phosphoimager (GE Healthcare).

### Western blot analysis

Flies of designated genotypes were raised at 25°C under standard conditions. Newly emerged adult flies were transferred into an incubator and entrained to LD 12∶12 at 30.5°C for 3 full days and then flash froze in liquid Nitrogen at the appropriate zeitgeber times on day 4. Heads of the frozen flies were harvested and ground into fine powder in liquid Nitrogen. The frozen powder was mixed with a mild lysis buffer (100 mM KCl, 5 mM MgCl2, 10 mM HEPES PH 7.0, 0.5% IGEPAL-CA630, 1 mM DTT, 1 mM PMSF, and 10 µg/ml protease inhibitor cocktail (Sigma), incubated on ice for 5 minutes, and centrifuged at 14,000× g for 20 minutes at 4°C. Cleared tissue lysate was obtained after the centrifugation and the concentration of total protein was determined. Approximately 10 ug samples of total protein were loaded onto 12% polyacrylamide gels. Electrophoresis and western blotting were carried out according to standard protocols. The DBT proteins were detected using anti-DBT antibodies provided by Dr. Jeffrey Price (University of Missouri-Kansas City).

### Translating ribosome affinity purification (TRAP)

Flies carrying the uas-EGFP-L10a construct [55] were crossed to appropriate *gal4* lines to express GFP-tagged ribosomes in desired cell types. Details of the TRAP method are described in [55]. Briefly, fly tissues were homogenized in a buffer containing 20 mM HEPES-KOH (pH 7.4), 150 mM KCl, 5 mM MgCl_2_, 10 µg/ml protease inhibitor cocktail (Sigma), 0.5 mM DTT, 20 unit/µl SUPERase.In RNase inhibitor (Invitrogen), and 100 µg/ml cycloheximide. Thirty mM DHPC and 1% IGEPAL-CA630 were added to the cleared tissue lysates. The mixtures were incubated on ice for 5 minutes and cleared again by centrifuging at 14,000× g for 20 minutes. The cleared lysates were applied to magnetic beads covered by purified anti-EGFP antibodies and incubated at 4°C with gentle rotating for 1 hour. After the IP, the beads were washed with a buffer containing 20 mM HEPES-KOH, pH 7.4, 5 mM MgCl_2_, 350 mM KCl, 1% IGEPAL-CA630, 0.5 mM DTT and 100 µg/ml cycloheximide. RNAs were extracted from the beads using the Trizol-LS Reagent (Invitrogen).

### Quantitative realtime PCR

Total RNA samples were treated with DNase I (Invitrogen) to eliminate potential contamination with genomic DNA. RNAs isolated from TRAP experiments were used directly since these RNAs usually do not carry genomic DNA contamination. Treated total RNAs or TRAP RNAs were primed with random hexamers (Ambion) and reverse transcribed into cDNAs using the Superscript II reverse transcriptase (Invitrogen). Quantification of the relative abundance of specific transcripts in the cDNA samples was conducted by Q-RT-PCR using 2X SYBR green PCR Master Mix (Applied Biosystems) and specific primers. Data were collected with Strategene Mx3000 or Mx4000. A pair of primers specific for the Ribosomal Protein 49 (Rp49) gene, which is known to be transcribed and translated at a constant rate throughout the circadian cycle (Huang and Jackson, unpublished observation), was used as an internal reference to account for variation in the input cDNA amount. Sequences for specific primers were: Rp49-F: GCCCAAGATCGTGAAGAAGC, Rp49-R: CGACGCACTCTGTTGTCG, *dbt*-*RA*-F: GATGCAAAACAACCCTTCGAATAC, *dbt*-*RA*-R: CCCAGGCGATATTTGTTACC, *dbt*-*RB*-F: AACGTAAGTGTCGAATTAGAAG, *dbt*-*RB*-R: CTGGCACTGTCCTTTCGTCT, *dbt*-*RC*-F: GCGACTGTGGCAACTACAAC, *dbt*-*RC*-R: CTGGCACTGTCCTTTCGTCT, *dbt*-*RE*-F: CGCTGCAGATGCGATAAAAA, *dbt*-*RE*-R: GATTTGCGTTGCCTTTCTGG.

### Behavioral analyses

Locomotor activity was assayed using 2- to 3-day-old males and the Drosophila Activity Monitoring (DAM) system (Trikinetics, Waltham, MA). Flies were loaded into activity monitors and placed in incubators set at either 30°C (for flies carrying *Tub-gal80^ts^*) or 23°C (for flies not carrying *Tub-gal80^ts^*), they were entrained to LD 12∶12 for 4–5 days and then released into constant darkness (DD) for an additional 7–10 days. Visualization of actograms and the analysis of rhythmicity and period length were performed using a signal processing toolbox [76] within the MATLAB software package (MathWorks). The toolbox analyzes circadian rhythmicity of fly locomotor activity by applying an autocorrelation analysis. The Rythmicity Index (RI) is defined as the height of the third peak in the correlogram resulting from this analysis (counting the peak at lag 0 as the first peak). Period length is determined by Fourier analysis [76]. Flies were considered rhythmic if they had a high RI value (generally greater than 0.2) as well as obvious rhythmicity by visual inspection of the actogram.

### Immunohistochemistry

To visualize PER cycling in the PDF neurons, adult flies were harvested at appropriate circadian times and fixed in 4% paraformaldehyde solution. Brains were dissected from the heads and washed in PBS and PBS-T (0.05% Triton X-100). For assessing LARK abundance in PDF neurons, adult flies were harvested at ZT 2 and brains were dissected prior to fixation. After dissection, the brains were fixed in 4% paraformaldehyde solution and then washed in PBS and PBS-T. Immunohistochemistry was carried out according to standard procedure for staining whole mount fly brains. Primary antibodies were used at the following dilutions: Rabbit anti-PER (1∶10000, R. Stanewsky), mouse anti-PDF (1∶10, DSHB), Rabbit anti-LARK (1∶1000, [47]). Secondary antibodies, goat anti-mouse IgG (Alexa-488 conjugated, Molecular Probes) and goat anti-rabbit (Cy3 conjugated or Alexa-488 conjugated, Molecular Probes) were used at a dilution of 1∶300 and an incubation time of at least 5 hours. Confocal images were acquired from brain whole mounts using a Leica TCS SP2 AOBS microscope within the Tufts Center for Neuroscience Research (CNR) Imaging Core. Blind scoring for PER nuclear versus cytoplasmic localization in the s-LNvs was accomplished by using the following scoring system: 0 = no staining in nuclei, 1 = mixture of nuclear and cytoplasmic staining, and 2 = nuclear staining only. To assess the time course of PER degradation in the nuclei of l-LNvs, a custom ImageJ macro program was used to quantify PER immunoreactivity. All l-LNvs in a brain hemisphere of a particular animal were imaged as a 3D stack with optical sections in 1 µm steps under a 63× oil lens objective. The section with the largest cell diameter, i.e. the middle section of the cell, was identified and an ROI was drawn manually outlining the nucleus. Average pixel intensity within the ROI was calculated for each individual l-LNv cell in a brain hemisphere. The value obtained for individual cells were then further averaged among all cells in a same brain hemisphere to get a value for each individual animal.

## Supporting Information

Figure S1 Different versions of genome annotation of the *dco* (i.e. *dbt*) region by Flybase. A, Previous annotation from Release 5.3, showing only three alternative transcripts, *dco-RA*, *dco-RB*, and *dco-RC*. B. Current annotation from Release 5.49, showing an additional transcript named *dco-RD* with extended 3′UTR. C. Existing EST sequences aligning to the region of the genome. The two ESTs supporting our annotation of an additional 5′ variant, which we called “*dbt-RE*”, are indicated by arrows. Location of primers used to specifically amplify individual dbt transcripts in Q-RTPCR experiments are indicated by red arrows. The forward primers for RA and RB each span a splice junction, thus were drawn across the respective introns, although their sequences does not include any intronic sequence.
(TIF)

Figure S2 Increased LARK expression has minimal effect on the abundance of *dbt* transcripts in total RNA extracts. Average fold change in transcript abundance in total RNA samples isolated from LARK OE versus control animals is shown for each transcript. (n = 6, including 3 biological replicates with 2 technical replicates each; error bars represent the possible range of change calculated based on SEM, * p<0.02 Student's t-test).
(TIF)

Figure S3 The abundances of *dbt-RC* and *dbt-RE* in total RNA extracted from wild-type flies do not exhibit circadian changes. A. Abundance profile of *dbt-RC* in the first day of DD. B. Abundance profile of *dbt-RE* in LD. Abundances in the time series are normalized to that of the first time point. n = 6 (2 biological replicates, each with 3 technical replicates) for all data points; error bars represent the possible range of fold change calculated based on SEM.
(TIF)

Figure S4 The period-shortening effect of LARK KD can be reverted by increasing either LARK or DBT level. Genotypes shown are: *w^1118^*; *pdf-gal4 uas-dicer2/+; lark^1^ uas-lark^RNAi^/+* (alone, n = 11), *w^1118^*; *pdf-gal4 uas-dicer2/+; lark^1^ uas-lark^RNAi^/uas-lark* (with uas-lark, n = 26), *w^1118^*; *pdf-gal4 uas-dicer2/+; lark^1^ uas-lark^RNAi^/uas-dbt* (with uas-dbt, n = 59), Error bars represent SEM. *** p<10^−9^ based on Student's t-test.
(TIF)

Figure S5 LARK OE delays, whereas LARK KD accelerates PER cycling in the s-LNv neurons under free-running conditions. A–B, Representative images showing PER immunoreactivity at various circadian times (CTs) during DD day 4 in the s-LNvs of LARK OE, overexpression control (OC), LARK KD, and KD control (KC) flies. Genotypes for OE, OC, KD and KC are the same as those shown in Figure 5. C, Quantification of results from two independent experiments by blind scoring of PER using the following system: 0 = no nuclear staining, 1 = mixture of nuclear and cytoplasm staining, 2 = nuclear staining only. Each individual image was scored by two different observers and the two scores were then averaged. Scores of all images for the same genotype at the same time point were averaged and plotted.
(TIF)

Figure S6 Western blot showing effect of altered LARK level on the expression of DBT protein. OE: overexpression. OC: control for overexpression. Times of sample collections (ZT2 or ZT14) are indicated. Overexpression of LARK or DBT was achieved by driving *uas-lark* or *uas-dbt* with *elav-gal4*. Higher molecular weight DBT-immunoreactive bands can be visualized with DBT OE on a longer exposure of the blot. Black arrow: known DBT isoform. Red arrow: novel short DBT isoform. Arrow head: high molecular weight DBT-immunoreactive bands. *: a non-specific band serving as a loading control. Molecular weight standards (in KD) are shown on the left side of the image.
(TIF)

Figure S7 Western blot showing that LARK overexpression does not induce the smaller isoform at pupal stage. OE: overexpression. OC: control for overexpression. Time of sample collections are indicated. Overexpression of LARK was achieved by driving *uas-lark* with *elav-gal4*. Samples extracted from whole pupae are on the left (lanes 1–4), sample extracted from adult heads (as a positive control) are on the right (lanes 6–7). Lane 5: molecular weight ladder. Black arrow: known DBT isoform. Red arrow: novel short DBT isoform (only seen in adult head OE sample). Upper and lower panels show the same blot with different exposure times. Exposure time in the lower panel was reduced to allow a clear view of the novel short DBT isoform in the adult OE sample.
(TIF)

Table S1 Period and Rythmicity Index (RI) for all characterized genotypes. Abbreviated labels are the same as those used in Figures. The table shows genotypes, number of flies tested, the rhythmic fraction of flies tested, rhythmicity index, and circadian period.
(DOCX)
