# Supplementary figures and images for: Translational Regulation of the DOUBLETIME/CKIδ/ε Kinase by LARK Contributes to Circadian Period Modulation
Source: PLoS Genet. 2014 Sep 11;10(9):e1004536. doi: 10.1371/journal.pgen.1004536 (PMC4161311; doi:10.1371/journal.pgen.1004536)

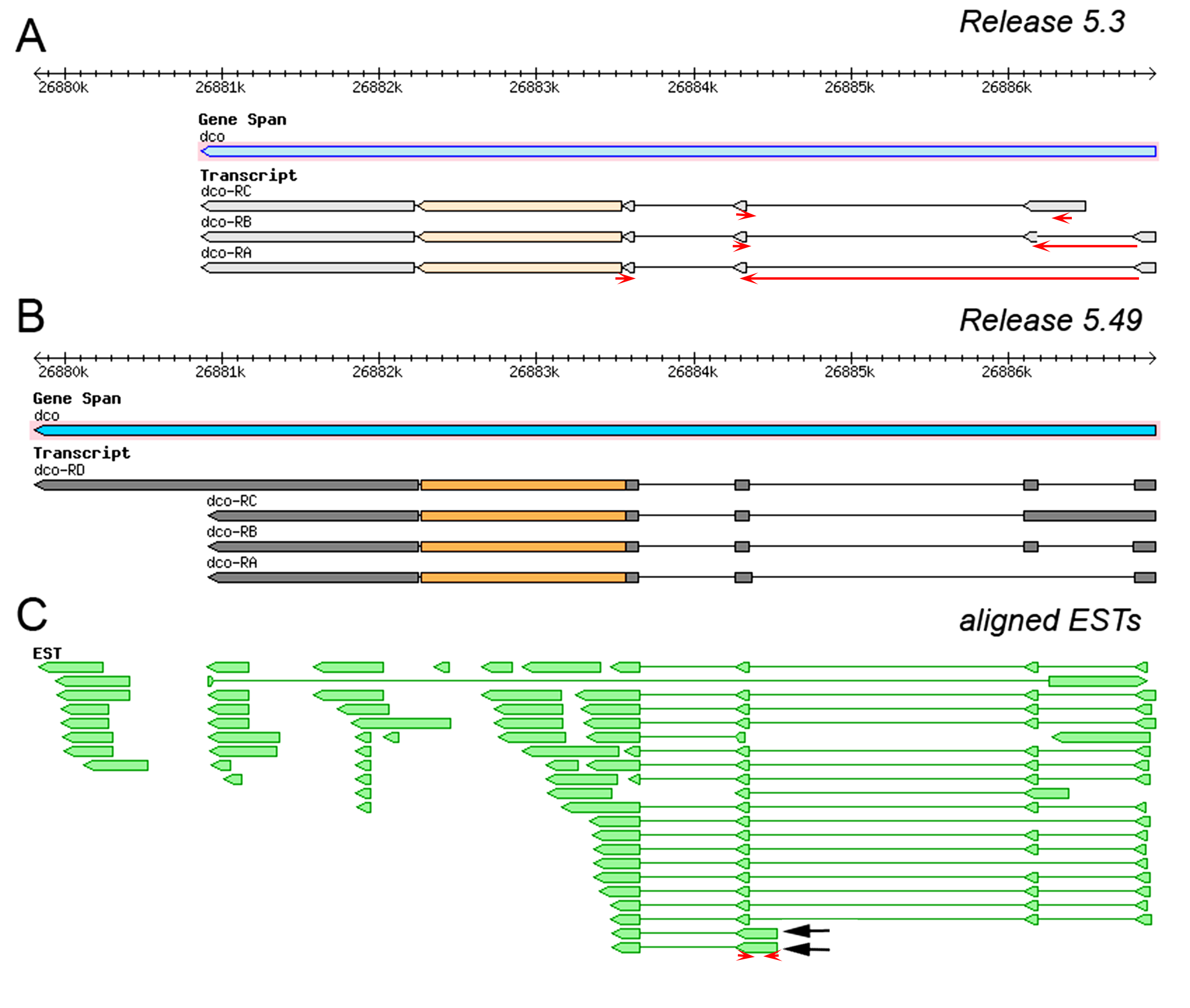

Supplement: Figure S1 — Different versions of genome annotation of the dco (i.e. dbt) region by Flybase. A, Previous annotation from Release 5.3, showing only three alternative transcripts, dco-RA, dco-RB, and dco-RC. B. Current annotation from Release 5.49, showing an additional transcript named dco-RD with extended 3′UTR. C. Existing EST sequences aligning to the region of the genome. The two ESTs supporting our annotation of an additional 5′ variant, which we called “dbt-RE”, are indicated by arrows. Location of primers used to specifically amplify individual dbt transcripts in Q-RTPCR experiments are indicated by red arrows. The forward primers for RA and RB each span a splice junction, thus were drawn across the respective introns, although their sequences does not include any intronic sequence. (TIF) [file pgen.1004536.s001.tif]

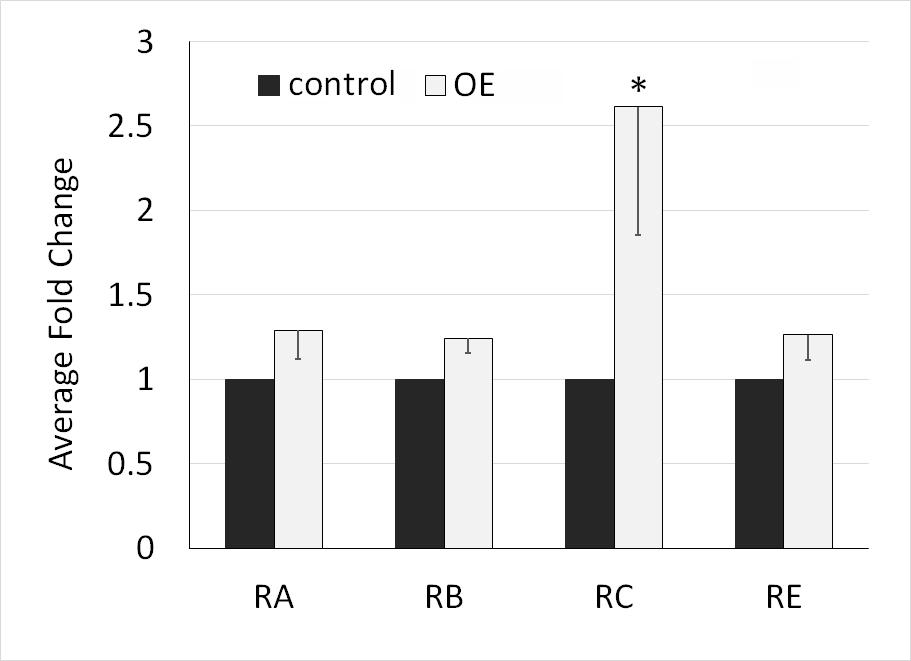

Supplement: Figure S2 — Increased LARK expression has minimal effect on the abundance of dbt transcripts in total RNA extracts. Average fold change in transcript abundance in total RNA samples isolated from LARK OE versus control animals is shown for each transcript. (n = 6, including 3 biological replicates with 2 technical replicates each; error bars represent the possible range of change calculated based on SEM, * p<0.02 Student's t-test). (TIF) [file pgen.1004536.s002.tif]

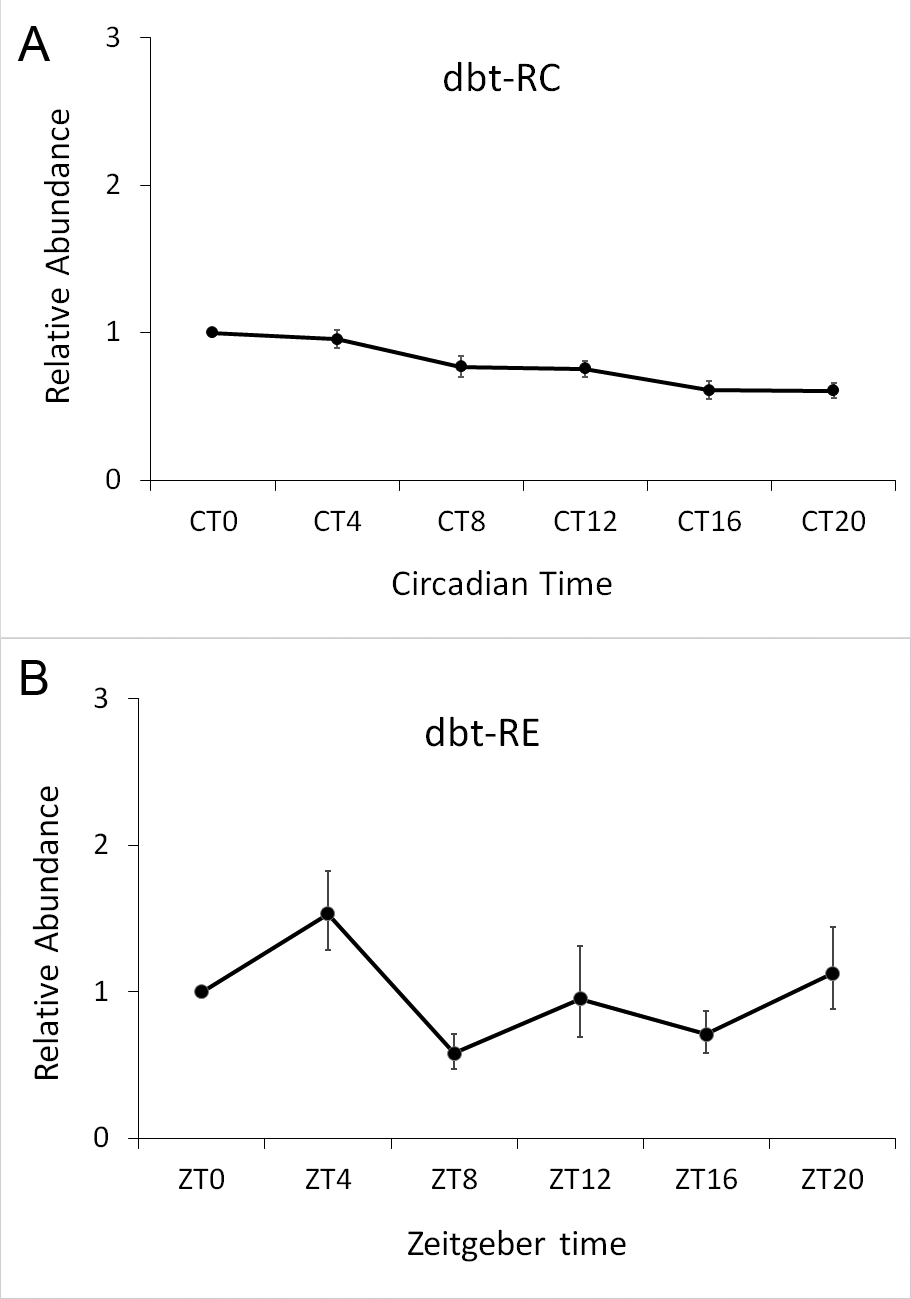

Supplement: Figure S3 — The abundances of dbt-RC and dbt-RE in total RNA extracted from wild-type flies do not exhibit circadian changes. A. Abundance profile of dbt-RC in the first day of DD. B. Abundance profile of dbt-RE in LD. Abundances in the time series are normalized to that of the first time point. n = 6 (2 biological replicates, each with 3 technical replicates) for all data points; error bars represent the possible range of fold change calculated based on SEM. (TIF) [file pgen.1004536.s003.tif]

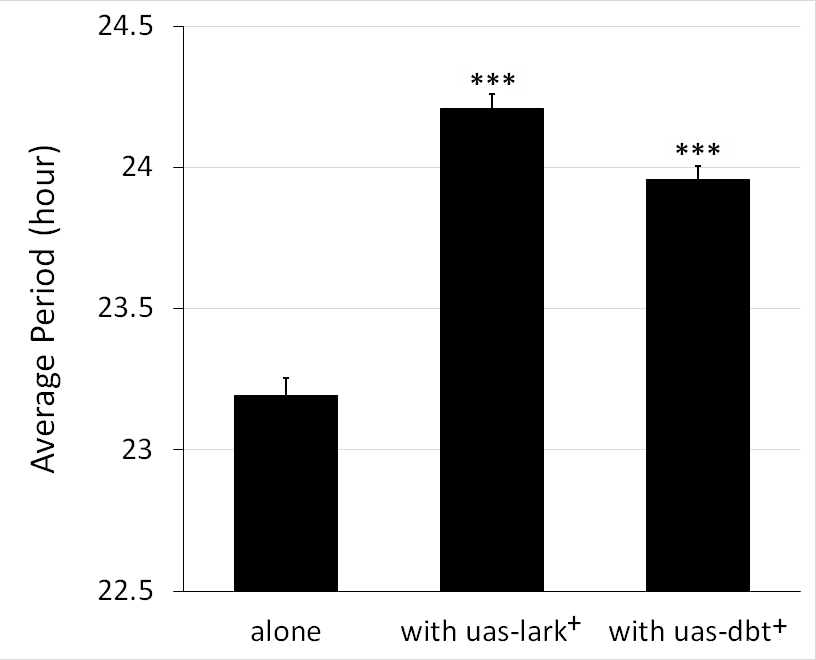

Supplement: Figure S4 — The period-shortening effect of LARK KD can be reverted by increasing either LARK or DBT level. Genotypes shown are: w1118; pdf-gal4 uas-dicer2/+; lark1 uas-larkRNAi/+ (alone, n = 11), w1118; pdf-gal4 uas-dicer2/+; lark1 uas-larkRNAi/uas-lark (with uas-lark, n = 26), w1118; pdf-gal4 uas-dicer2/+; lark1 uas-larkRNAi/uas-dbt (with uas-dbt, n = 59), Error bars represent SEM. *** p<10−9 based on Student's t-test. (TIF) [file pgen.1004536.s004.tif]

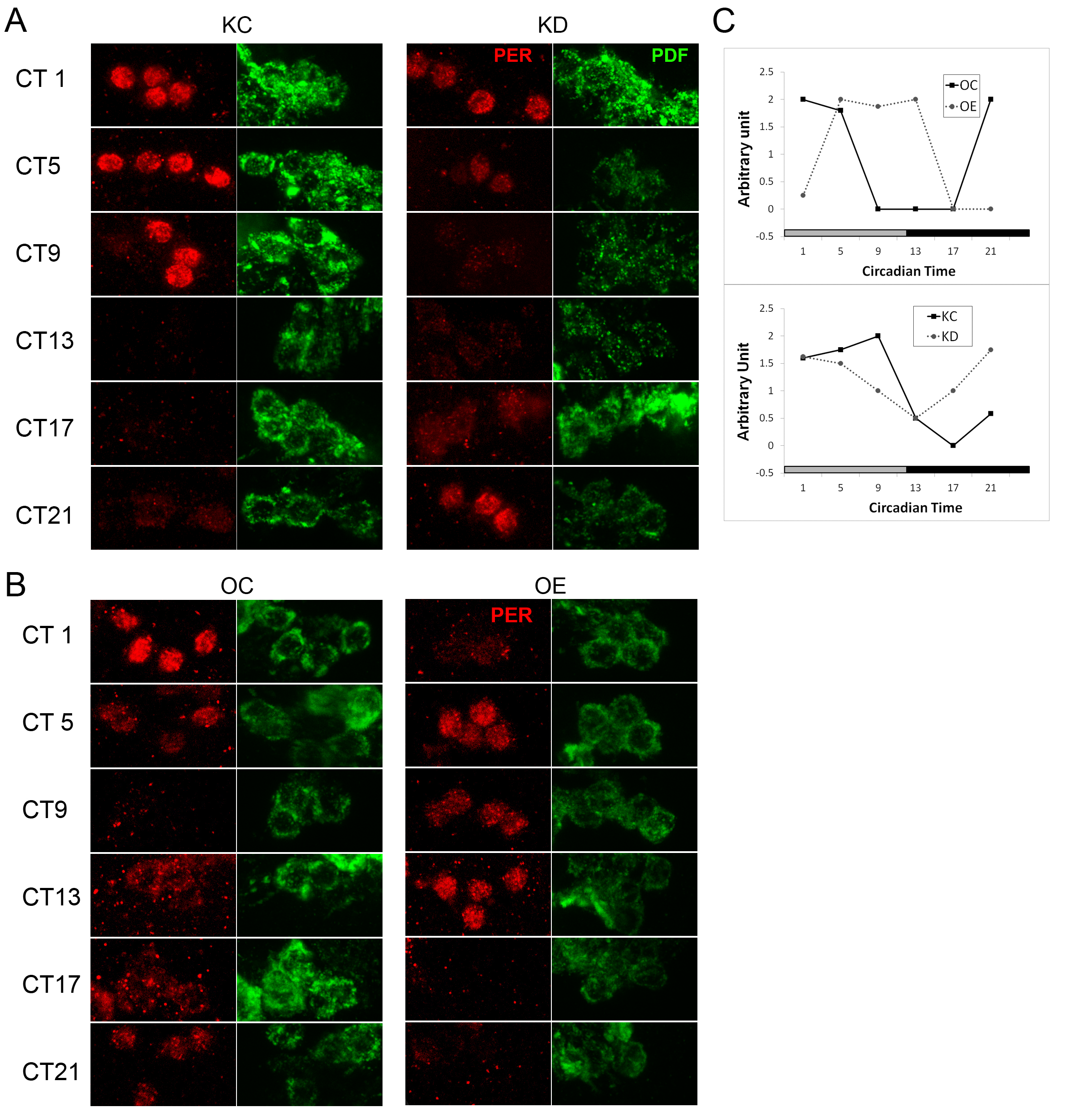

Supplement: Figure S5 — LARK OE delays, whereas LARK KD accelerates PER cycling in the s-LNv neurons under free-running conditions. A–B, Representative images showing PER immunoreactivity at various circadian times (CTs) during DD day 4 in the s-LNvs of LARK OE, overexpression control (OC), LARK KD, and KD control (KC) flies. Genotypes for OE, OC, KD and KC are the same as those shown in Figure 5. C, Quantification of results from two independent experiments by blind scoring of PER using the following system: 0 = no nuclear staining, 1 = mixture of nuclear and cytoplasm staining, 2 = nuclear staining only. Each individual image was scored by two different observers and the two scores were then averaged. Scores of all images for the same genotype at the same time point were averaged and plotted. (TIF) [file pgen.1004536.s005.tif]

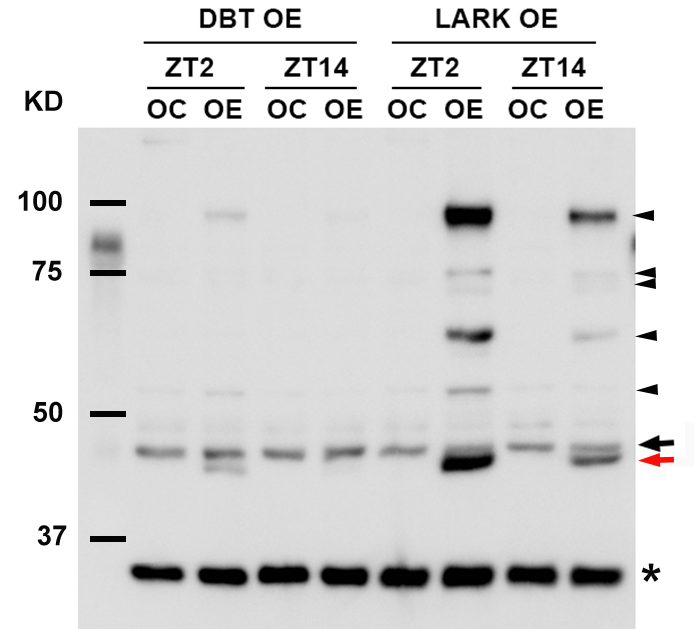

Supplement: Figure S6 — Western blot showing effect of altered LARK level on the expression of DBT protein. OE: overexpression. OC: control for overexpression. Times of sample collections (ZT2 or ZT14) are indicated. Overexpression of LARK or DBT was achieved by driving uas-lark or uas-dbt with elav-gal4. Higher molecular weight DBT-immunoreactive bands can be visualized with DBT OE on a longer exposure of the blot. Black arrow: known DBT isoform. Red arrow: novel short DBT isoform. Arrow head: high molecular weight DBT-immunoreactive bands. *: a non-specific band serving as a loading control. Molecular weight standards (in KD) are shown on the left side of the image. (TIF) [file pgen.1004536.s006.tif]

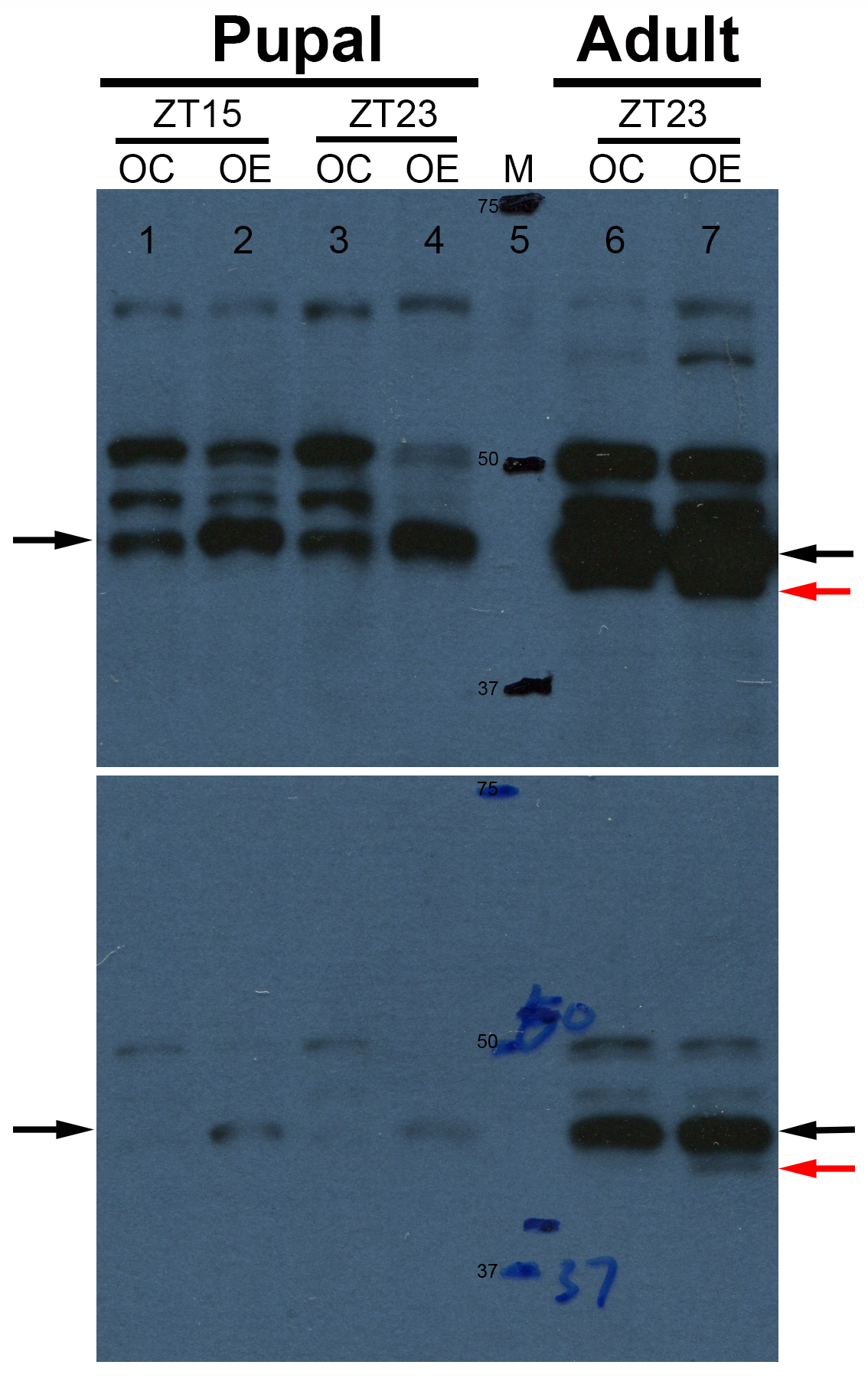

Supplement: Figure S7 — Western blot showing that LARK overexpression does not induce the smaller isoform at pupal stage. OE: overexpression. OC: control for overexpression. Time of sample collections are indicated. Overexpression of LARK was achieved by driving uas-lark with elav-gal4. Samples extracted from whole pupae are on the left (lanes 1–4), sample extracted from adult heads (as a positive control) are on the right (lanes 6–7). Lane 5: molecular weight ladder. Black arrow: known DBT isoform. Red arrow: novel short DBT isoform (only seen in adult head OE sample). Upper and lower panels show the same blot with different exposure times. Exposure time in the lower panel was reduced to allow a clear view of the novel short DBT isoform in the adult OE sample. (TIF) [file pgen.1004536.s007.tif]
