# Supplementary material for: Translational Regulation of the DOUBLETIME/CKIδ/ε Kinase by LARK Contributes to Circadian Period Modulation
Source: PLoS Genet. 2014 Sep 11;10(9):e1004536. doi: 10.1371/journal.pgen.1004536 (PMC4161311; doi:10.1371/journal.pgen.1004536)
Supplement: Table S1 — Period and Rythmicity Index (RI) for all characterized genotypes. Abbreviated labels are the same as those used in Figures. The table shows genotypes, number of flies tested, the rhythmic fraction of flies tested, rhythmicity index, and circadian period. (DOCX) [file pgen.1004536.s008.docx]

| **Label in Figure** | **Genotype** | **N** | **% rhythmic** | **RI**  (mean ± SEM) | **Period (h)**  (mean ± SEM) |
| --- | --- | --- | --- | --- | --- |
| KC | *w^1118^*; *pdf-gal4 uas-dicer2/+* | 166 | 87% | 0.48 ± 0.04 | 24.1 ± 0.02 |
| KD | *w^1118^*; *pdf-gal4 uas-dicer2/+; lark^1^ uas-lark^RNAi^/+* | 130 | 89% | 0.41 ± 0.04 | 23.3 ± 0.03 |
| KD with uas-lark | *w^1118^*; *pdf-gal4 uas-dicer2/+; lark^1^ uas-lark^RNAi^/uas-lark* | 26 | 100% | 0.39 ± 0.02 | 24.2 ± 0.05 |
| KD with uas-dbt | *w^1118^*; *pdf-gal4 uas-dicer2/+; lark^1^ uas-lark^RNAi^/uas-dbt* | 59 | 94% | 0.45 ± 0.06 | 24.0 ± 0.05 |
| OC | *w^1118^*; *pdf-gal4/+; Tub-gal80^ts^/+* | 144 | 87% | 0.40 ± 0.03 | 23.6 ± 0.03 |
| OE | *w^1118^*; *pdf-gal4/+; Tub-gal80^ts^ uas-lark/+* | 192 | 88% | 0.32 ± 0.01 | 25.1 ± 0.06 |
| OE^RRM^ | *w^1118^*; *pdf-gal4/uas-lark^RRM^* | 64 | 89% | 0.49 ± 0.06 | 23.9 ± 0.06 |
| dbt^S^ without lark KD | *w^1118^*; *pdf-gal4 uas-dicer2/+; +/dbt^S^* | 57 | 92% | 0.40 ± 0.02 | 22.1 ± 0.08 |
| dbt^S^ with lark KD | *w^1118^*; *pdf-gal4 uas-dicer2/+; lark^1^ uas-lark^RNAi^ /dbt^S^* | 53 | 87% | 0.39 ± 0.02 | 21.4 ± 0.07 |
| dbt^L^ without lark KD | *w^1118^*; *pdf-gal4 uas-dicer2/+; +/dbt^L^* | 62 | 97% | 0.46 ± 0.02 | 25.5 ± 0.06 |
| dbt^L^ with lark KD | *w^1118^*; *pdf-gal4 uas-dicer2/+; lark^1^ uas-lark^RNAi^ /dbt^L^* | 50 | 89% | 0.43 ± 0.02 | 24.7 ± 0.08 |
| dbt^P^ without lark KD | *w^1118^*; *pdf-gal4 uas-dicer2/+; +/dbt^P^* | 25 | 91% | 0.46 ± 0.02 | 23.8 ± 0.07 |
| dbt^P^ with lark KD | *w^1118^*; *pdf-gal4 uas-dicer2/+; lark^1^ uas-lark^RNAi^ /dbt^P^* | 17 | 74% | 0.34 ± 0.04 | 22.8 ± 0.08 |
| dbt^AR^ without lark KD | *w^1118^*; *pdf-gal4 uas-dicer2/+; +/dbt^AR^* | 31 | 100% | 0.63 ± 0.02 | 29.5 ± 0.03 |
| dbt^AR^ with lark KD | *w^1118^*; *pdf-gal4 uas-dicer2/+; lark^1^ uas-lark^RNAi^ /dbt^AR^* | 24 | 96% | 0.53 ± 0.03 | 28.1 ± |
| dbt^S^ without lark OE | *w^1118^*; *pdf-gal4/+; Tub-gal80^ts^/dbt^S^* | 32 | 100% | 0.69 ± 0.01 | 21.1 ± 0.03 |
| dbt^S^ with lark OE | *w^1118^*; *pdf-gal4/+; Tub-gal80^ts^ uas-lark/dbt^S^* | 22 | 82% | 0.32 ± 0.02 | 23.6 ± 0.14 |
| dbt^L^ without lark OE | *w^1118^*; *pdf-gal4/+; Tub-gal80^ts^/dbt^L^* | 20 | 80% | 0.58 ± 0.01 | 25.2 ± 0.12 |
| dbt^L^ with lark OE | *w^1118^*; *pdf-gal4/+; Tub-gal80^ts^ uas-lark/dbt^L^* | 26 | 86% | 0.32 ± 0.04 | 26.3 ± 0.07 |
| dbt^P^ without lark OE | *w^1118^*; *pdf-gal4/+; Tub-gal80^ts^/dbt^P^* | 32 | 100% | 0.62 ± 0.01 | 23.5 ± 0.04 |
| dbt^P^ with lark OE | *w^1118^*; *pdf-gal4/+; Tub-gal80^ts^ uas-lark/dbt^P^* | 32 | 84% | 0.29 ± 0.02 | 26.1 ± 0.10 |
| dbt^AR^ without lark OE | *w^1118^*; *pdf-gal4/+; Tub-gal80^ts^/dbt^AR^* | 47 | 100% | 0.63 ± 0.01 | 26.5 ± 0.03 |
| dbt^AR^ with lark OE | *w^1118^*; *pdf-gal4/+; Tub-gal80^ts^ uas-lark/dbt^AR^* | 48 | 100% | 0.56 ± 0.01 | 27.1 ± 0.05 |
| pdf>dbt^+^ alone | *w^1118^; pdf-gal4/+; uas-dbt^+^/+* | 31 | 90% | 0.40 ± 0.02 | 23.9 ± 0.11 |
| pdf>*dbt*^+^ with lark OE | *w^1118^; pdf-gal4/+; uas-dbt^+^/ Tub-gal80^ts^ uas-lark* | 31 | 13% | 0.14 ± 0.02 | Arrhythmic |
| pdf>dbt^D132N^ alone | *w^1118^; pdf-gal4/+; uas-dbt^D132N^/+* | 42 | 100% | 0.47 ± 0.02 | 23.1 ± 0.06 |
| pdf>dbt^D132N^ with lark OE | *w^1118^; pdf-gal4/+; uas-dbt^D132N^/ Tub-gal80^ts^ uas-lark* | 14 | 93% | 0.38 ± 0.04 | 22.7 ± 0.11 |

**Table S1. Period and Rythmicity Index (RI) for all characterized genotypes.**
